# The Evolution of Automated Medical Billing With Artificial Intelligence: A Review With a Global and Saudi Perspective

**DOI:** 10.7759/cureus.96464

**Published:** 2025-11-10

**Authors:** Layla Khaled Nasser

**Affiliations:** 1 Department of Business Administration, Saudi Electronic University, Jeddah, SAU

**Keywords:** administrative costs, artificial intelligence, financial stability, health accounting, healthcare system, hospital administration, machine learning, medical billing, natural language processing

## Abstract

Medical billing is a complex and evolving aspect of healthcare administration that requires accurate documentation and efficient reimbursement processes to maintain institutional financial sustainability. This review explores how artificial intelligence (AI) is transforming medical billing through automation, error reduction, and fraud detection, with emphasis on global developments and initiatives in Saudi Arabia. A comprehensive review of recent studies and real-world applications was conducted to assess AI-driven innovations in automated coding, claims processing, and financial management. AI-based platforms have demonstrated substantial reductions in coding errors and faster claim turnaround times. AI algorithms trained on large billing datasets identify discrepancies in submitted claims, detect potential fraud, and recommend corrective actions, enhancing transparency and compliance. Automated coding systems translate clinical documentation into standardized billing terminology, a global coding framework that assigns numeric identifiers to medical procedures and services, thus improving consistency and reducing human error. Despite these advancements, challenges remain, including data privacy, algorithmic bias, and infrastructure limitations. In conclusion, integrating AI into medical billing can revolutionize administrative efficiency, reduce operational costs, and promote financial sustainability across healthcare systems worldwide, including Saudi Arabia.

## Introduction and background

Business intelligence involves collecting, analyzing, and interpreting large volumes of data using advanced technologies and methodologies to generate meaningful insights that drive strategic decision-making within medical organizations [1]. In the context of medical billing, this capability is particularly valuable, as the field is characterized by intricate coding systems and a constantly evolving reimbursement framework, which pose significant challenges for healthcare systems globally.

Financial sustainability in healthcare depends on accurate and efficient billing practices, yet complexities such as coding errors, reimbursement delays, and elevated administrative costs frequently arise [1]. These issues stem from navigating a web of regulations, payer guidelines, and diverse coding systems, which vary across private insurers and government programs. Administrative costs, particularly those associated with insurance processing and billing operations, account for a substantial and increasing share of healthcare expenditures. One labor-intensive example is coding charts for billing, a manual, repetitive, and costly process. These administrative expenses represent nearly 20% of total healthcare spending, equating to approximately $600 billion spent annually in the United States [2]. Specific data on administrative costs in Saudi Arabia's healthcare system are limited, but general insights into healthcare expenditures are available [2]. In 2021, Saudi Arabia's healthcare spending reached 6% of gross domestic product (GDP), with 77% from public funds. Unlike the United States, where administrative costs comprise 20% of expenditures, equivalent data for Saudi Arabia is unavailable.

Frequent updates to coding requirements and the transition from fee-for-service to value-based care models, which emphasize quality and outcomes, add further administrative burdens on providers. Meeting value-based standards necessitates detailed data reporting and adjustments in billing processes to ensure compliance and demonstrate performance in achieving quality metrics [3,4]. Addressing these challenges requires innovative solutions such as artificial intelligence (AI)-powered technologies. Tools like natural language processing (NLP) and machine learning (ML) offer immense potential to streamline coding, reduce errors, and optimize billing efficiency [5]. By combining business intelligence with AI, healthcare organizations can improve operational efficiency, enhance financial sustainability, and adapt to the complex medical billing landscape.

This review examines the applications, benefits, and challenges of AI integration in medical billing, addressing a key gap in existing literature. While prior studies focus broadly on AI in healthcare, few explore its direct use in automated billing, fraud detection, and financial optimization. Additionally, comparative insights between global developments and Saudi Arabia’s rapidly evolving healthcare system remain limited. This review bridges that gap by synthesizing evidence from healthcare informatics, finance, and AI engineering to present a practical understanding of AI-driven billing, emphasizing cost efficiency, accuracy, and regulatory implications within modern healthcare and Vision 2030 transformation goals [6].

Methodology

To ensure transparency, reproducibility, and scientific rigor, this narrative review was conducted to examine the evolution of automated medical billing through AI integration in healthcare systems. A comprehensive literature search was performed across PubMed, Scopus, and Web of Science databases up to July 2025. The search strategy utilized Boolean operators (AND/OR) to combine relevant keywords, including “Artificial Intelligence”, “Machine Learning”, “Medical Billing”, “Healthcare Finance”, “Automation”, “Fraud Detection”, and “Revenue Cycle Management”.

Eligible publications included peer-reviewed original research articles, systematic and narrative reviews, case studies, and institutional reports published in English that explored AI applications in medical billing, coding automation, fraud detection, claim management, or financial optimization. Titles and abstracts were screened for relevance, followed by full-text review. Studies focusing exclusively on general AI in medicine without a specific connection to billing or administrative efficiency were excluded.

All selected studies were qualitatively analyzed to extract information on AI methodologies (e.g., ML, NLP, predictive analytics), practical outcomes, challenges, and regional applications, particularly within the Saudi healthcare system. The synthesis emphasized comparative insights, evidence strength, and practical implications for healthcare finance.

Given the narrative nature of this review, no quantitative or meta-analytic methods were applied; instead, the findings were thematically categorized into domains addressing automation efficiency, fraud detection, revenue optimization, and implementation challenges.

## Review

AI refers to the simulation of human cognitive functions, such as learning, reasoning, and problem-solving, by machines, enabling them to perform tasks that traditionally require human intelligence [7]. ML, a key subset of AI, develops algorithms that learn from data and improve performance without explicit programming. While AI encompasses the broader concept of intelligent automation, ML provides the data-driven foundation that powers predictive and analytical capabilities. Within healthcare administration, AI-driven ML models are increasingly applied to automate billing processes, optimize medical coding, and enhance administrative efficiency. These systems analyze electronic health records (EHRs), detect coding inconsistencies, predict claim rejections, and streamline reimbursement workflows, reducing human error and financial loss [8].

Rather than replacing healthcare professionals, AI complements their expertise by facilitating faster, more accurate administrative decision-making and supporting the financial sustainability of healthcare systems. AI in medicine is broadly classified into three main categories: virtual, physical, and hybrid systems, where robots collaborate within virtual environments [9]. These AI-driven technologies assist physicians by providing medical insights, managing administrative tasks, and optimizing healthcare business operations. A widely recognized application is ChatGPT (OpenAI, San Francisco, California, United States), a generative AI model that utilizes deep learning algorithms to generate natural-sounding text. Users can interact with ChatGPT via text-based or voice input interfaces [10]. The software is available online, offering both a subscription-based version with advanced features and a free version with limited capabilities.

Liu et al. highlighted three key clinical areas where generative AI can improve medical documentation: patient clinic letters, medical notes, and radiology reports [10]. In radiology, deep learning algorithms have enhanced the detection of complex patterns, providing radiologists with advanced tools to support accurate decision-making when analyzing imaging data, such as conventional radiographs. Jeblick et al. demonstrated that generative AI can also simplify radiology reports for patients while maintaining high accuracy [11].

AI has been increasingly utilized to collect patient data, not only to assist in clinical decision-making but also to generate health risk alerts and streamline patient services [12,13]. In the healthcare industry and business sector, AI is now an integral component of business intelligence, supporting medical coding, billing, and pricing strategies, risk management, and innovations in treatment procedures [14]. In recent years, policymakers, public health officials, and researchers have intensified their focus on public health financing challenges. The high expansion of healthcare data, influenced by demographics, diagnoses, and comorbidities, has led to complex and unpredictable financial systems [15]. AI-driven healthcare management tools are playing a vital role in bridging the gap between limited resources and increasing public healthcare demands [16]. Ramezani et al. conducted a scoping review analyzing AI applications in healthcare financing from 2000 to 2023 [17]. Their findings highlight AI's role in governance, revenue raising, pooling, and strategic purchasing within health systems. The study recommends leveraging AI tools to optimize public health financing and provide better support for vulnerable populations globally.

Implementation of referencing tools such as Mendeley (Mendeley Ltd., London, United Kingdom) or Zotero (Corporation for Digital Scholarship, Vienna, Virginia, United States) enables healthcare institutions to gain a deeper understanding of their operations and competitive positioning through integrated data analysis, mining, visualization, and reporting [1]. AI enhances these business intelligence functions by automating data extraction, pattern recognition, and performance evaluation. Techniques such as NLP and computer vision allow the retrieval of valuable information from unstructured data sources, including text documents, images, and videos. The incorporation of ML within business intelligence systems delivers transformative advantages, improving accuracy, efficiency, and strategic decision-making within healthcare organizations [18]. These technologies help automate labor-intensive processes such as data cleansing, integration, and report generation, thereby reducing manual workload and optimizing resource allocation. It also enhances data analysis by identifying complex patterns and trends that may be missed by human analysts, enabling more accurate forecasting and data-driven decision-making. Additionally, these technologies support personalized insights by analyzing clients/patients’ behaviors and preferences, leading to improved patient experiences [19].

AI and medical billing

**Figure 1:**
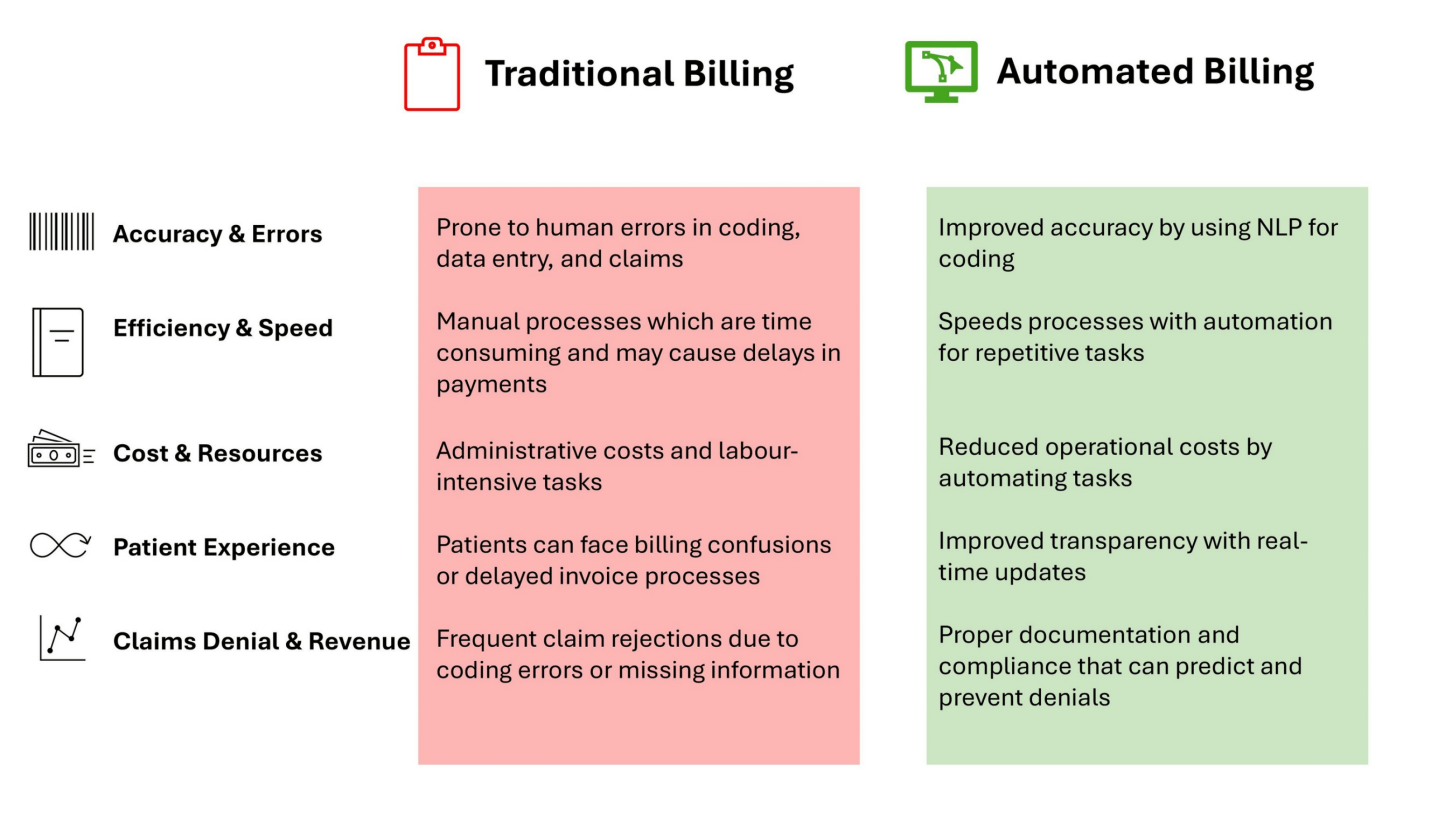
Difference between traditional and automatic medical billing. Image Credit: Author NLP: natural language processing

The traditional medical billing system relies extensively on manual documentation, where coders must enter diagnostic and procedural codes individually into printed or electronic forms, rendering the process labor-intensive, error-prone, and time-consuming [1]. Paper-based billing involves multiple steps, from submission to medical billing organizations to payer approval, leading to delays and administrative burdens. However, AI automation changes this routine system and enhances accuracy and efficiency, transforming medical billing and coding into a digitalized system [20,21]. AI-powered pricing algorithms adjust billing rates dynamically based on market conditions, patient demographics, and service demand, while also automating claim processing, coding assistance, and fraud detection (Figure 1).

Dynamic pricing utilizes real-time data to modify charges for services such as imaging and elective procedures. AI algorithms assess pricing elasticity by evaluating historical pricing trends and patient responses to determine optimal billing thresholds. This balance ensures revenue generation while maintaining accessibility for patients. By analyzing competitor pricing, consumer behavior, and industry trends, hospitals and clinics refine their billing strategies, strengthen their market position, and promote financial sustainability [18].

AI has significantly enhanced healthcare operations, with a notable report in 2013 estimating potential annual cost savings of approximately $350 billion [22]. It has been widely applied in plastic surgery for diagnosis generation, outcome prediction, personalized treatment planning, and improving billing codes for procedures [23]. These advancements primarily focus on optimizing healthcare billing to minimize revenue loss, streamline payments and reimbursements, and enhance patient satisfaction. The billing process relies on clinical documentation from hospitals and clinics, where codes are assigned to indicate the complexity of services provided. These codes are then used by billing staff to process insurance claims or generate invoices for patients [24]. Extracting, editing, auditing, and finalizing payments in healthcare billing is labor-intensive and heavily dependent on coding personnel. Physician documentation is transformed into billable monetary values through a process known as charge entry, wherein clinical services are systematically translated into Current Procedural Terminology (CPT) codes to facilitate standardized billing, accurate reimbursement, and compliance with healthcare regulatory frameworks [25].

The CPT system, developed by the American Medical Association (AMA), establishes a standardized framework for coding medical, surgical, and diagnostic procedures, facilitating communication among healthcare providers [25]. CPT codes ensure billing consistency, allowing providers to document services accurately for insurance companies and patients. These codes streamline reimbursement processes, enhance documentation, and standardize billing practices across the United States healthcare system. To ensure compliance with billing regulations, CPT codes undergo multiple review stages, including claim editing and scrubbing, where errors are identified and corrected. Once reviewed, claims are submitted to insurance providers and finalized for payment. Accurate CPT coding is essential for efficient documentation and billing, as errors can significantly impact revenue [25]. Studies indicate coding errors of 38% in anesthesia, 80% in otolaryngology, 46% in general surgery, and 41% in orthopedic surgery [26,27]. A time-driven activity-based costing study found that medical billing takes an average of 75 minutes for ambulatory procedures and 100 minutes for inpatient procedures, with physicians spending approximately 15 minutes per case, equating to $50 per procedure [24]. To enhance efficiency, Reich et al. implemented an automated point-of-care electronic charge voucher system in an academic anesthesiology practice, resulting in a 3% increase in annual revenue and a 10-day reduction in accounts receivable [28].

**Figure 2:**
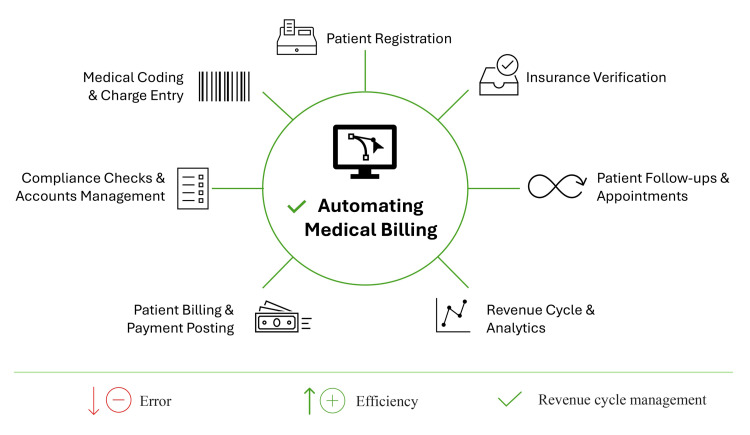
Application of automated medical billing system using artificial intelligence Image Credit: Author

Prior authorization is another significant challenge in medical billing and CPT coding, requiring physicians to provide clinical justification before insurers approve payment for medications or services. This process often causes delays and frustration for both patients and healthcare providers [25]. A survey by the AMA found that 90% of physicians consider prior authorization burdensome, frequently leading to delays in patient care [25]. AI technologies offer a promising solution by automating claims processing, reducing billing errors, and expediting authorization procedures. The use of AI in claims processing not only improves accuracy but also accelerates reimbursement by minimizing rejections and delays. Additionally, AI systems enhance processing speed, leading to significant time savings and increased efficiency in medical billing operations (Figure 2).

**Figure 3:**
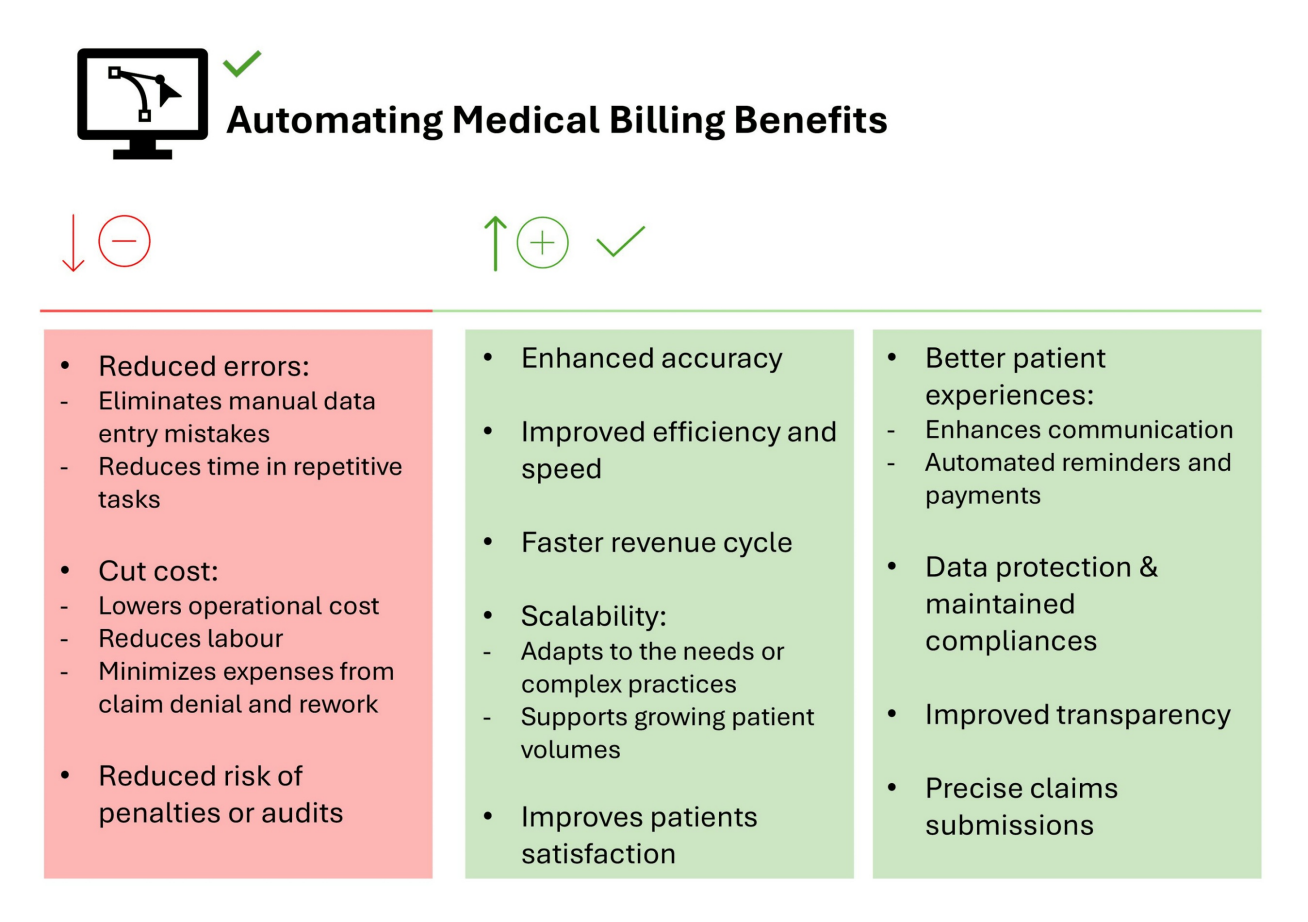
Benefits and disadvantages of automated medical billing Image Credit: Author

AI-powered technologies are increasingly utilized to enhance the integration of hospital billing, prior authorization, and medical coding, leading to improved outcomes. Accurate and efficient medical coding is essential for ensuring proper billing and reimbursement within the healthcare sector [29]. Incorporating AI in coding assistance offers several advantages (Figure 3). 

AI-driven systems accelerate the coding process by analyzing large volumes of documentation and generating coding suggestions significantly faster than manual methods. This efficiency is particularly valuable in healthcare settings, where timely billing is critical due to the continuous influx of patient records. Additionally, AI improves coding accuracy by processing complex medical data and providing precise recommendations that align with coding guidelines. This reduces errors, decreases claim rejections, and ensures regulatory compliance. By minimizing inaccuracies, AI helps healthcare organizations prevent financial losses and streamline reimbursement processes, ultimately enhancing operational efficiency and revenue cycle management [1,29].

The integration of AI-assisted coding can be enhanced through NLP, particularly for its ability to generate CPT codes from unstructured text within electronic medical records (EMRs) [24,30,31]. As a branch of ML, NLP is designed to analyze and interpret free-text data, allowing it to extract critical information from complex medical documents and suggest appropriate codes based on the provided documentation. It has gained significant attention in clinical medicine due to the high volume of unstructured text, which constitutes approximately 80% of medical records [32].

AI-powered tools utilizing NLP present opportunities to reduce hospital coding errors, improve accuracy, and optimize revenue across various medical specialties. Morey et al. developed an AI ensemble model incorporating NLP and ML to predict billing codes for 300,000 emergency department (ED) encounters, achieving high accuracy with an area under the curve (AUC) of 0.94-0.95 for levels 4 and 5 billing codes [33]. Automating ED coding has significantly reduced administrative costs and processing time. Similarly, Zaidat et al. demonstrated that the XLNet-based NLP model, a transformer architecture that improves upon BERT (Bidirectional Encoder Representations from Transformers) by capturing bidirectional and contextual word dependencies, was capable of automatically generating CPT codes from operative notes for spinal surgery procedures [34]. The model achieved high AU values of up to 0.95 and class-wise accuracy rates of up to 88%. Both studies underscore that inaccuracies in coding can delay reimbursements and result in significant financial losses, reinforcing the importance of AI-driven precision in optimizing billing efficiency. Furthermore, while these NLP models have demonstrated robust generalization in English-language datasets, their transferability to non-Western healthcare systems, such as Saudi Arabia, requires adaptation to local coding systems (e.g., International Statistical Classification of Diseases and Related Health Problems, Tenth Revision (ICD-10), Saudi billing regulations) and training on region-specific medical documentation to ensure comparable accuracy and compliance.

NLP progress in automating medical billing

NLP as an AI method enables machines to understand, interpret, and generate human language by converting unstructured clinical text into structured data for analysis. Prominent systems such as Columbia University’s Medical Language Extraction and Encoding (MedLEE), Brigham and Women’s Hospital’s Health Information Text Extraction (HITEx), and the Mayo Clinic’s Text Analysis and Extraction System (cTAKES) have been widely evaluated. MedLEE, for instance, demonstrated over 80% precision in early clinical text extraction but was validated on limited datasets of radiology reports [31], while HITEx and cTAKES expanded scalability but faced challenges in generalizability due to variability in documentation style and terminology across hospitals [35]. Despite their success, these systems highlight ongoing limitations in sample diversity, validation standardization, and adaptability to non-English datasets-factors essential for implementing NLP-driven automation in billing and coding [31,35].

These tools enhance the accuracy and efficiency of medical coding by automating the extraction of critical information from unstructured clinical data. NLP has also demonstrated its potential in generating CPT codes from operative notes. For example, Kim et al. conducted a retrospective analysis of elective spine surgery operative notes from 2015 to 2020, comparing CPT codes generated by the billing department with those produced by a deep learning NLP algorithm and a random forest ML model [30]. The random forest model achieved an accuracy of 87% compared to the senior billing coder, highlighting its effectiveness and reliability. Such advancements reduce dependency on manual coding, minimize errors, and expedite claims processing, leading to faster reimbursement. Greenburg et al. showcased AI's capability to detect incorrect coding [36]. They developed an open-source ML algorithm designed to interpret CPT codes from pathology reports and identify discrepancies compared to the original coder's work. The system then notifies the coder to reassess the codes, helping to ensure accuracy and preventing instances of underbilling. By leveraging deep learning and ML models, NLP can address the complexities of medical billing and improve workflow efficiency. These innovations not only reduce the workload for coding personnel but also ensure compliance with regulatory standards, optimize revenue cycles, and support scalability in healthcare billing systems.

As NLP tools continue to evolve, they hold great promise for enhancing accuracy and reliability across diverse medical specialties. Automated medical coding companies like Nym Health (New York, United States), CodaMetrix (Boston, Massachusetts, United States), and Fathom (San Francisco, California, United States) have developed NLP-based systems to enhance revenue cycle management. Nym Health achieves 96% accuracy in decoding provider notes from EMRs, generating ICD-10 and CPT codes within seconds, and providing traceable audit documentation [37]. This automation reduces physicians’ workload and allows coders to focus on complex cases. Currently, Nym Health handles coding for over 250 healthcare facilities worldwide and has expanded into outpatient visits [38]. CodaMetrix was developed to integrate deep ML and NLP techniques for automated assignment of diagnostic and procedural codes [39]. Similarly, Fathom uses deep learning and NLP to analyze EMR notes, generate ICD-10 and CPT codes, and recently partnered with Google Cloud Marketplace (Google LLC, Menlo Park, California, United States) to streamline revenue cycle processes [40]. Automated coding systems enhance revenue capture while reducing labor costs associated with manual coding. For example, Burns et al. developed ML models with NLP to assess the accuracy of AI-generated anesthesiology CPT codes, achieving 88% accuracy when matched against institution-assigned codes [27]. These models are now used to improve auditing and resubmission processes

Regarding prior authorization for hospital-based patient services, insurance providers are increasingly implementing AI-driven systems to streamline approval workflows. One insurer, in collaboration with a technology firm, piloted such a system [34]. By cross-referencing EMRs with predefined eligibility requirements, the system delivered real-time responses to physicians, reducing the average approval time from nine days to just one. Moreover, it electronically processed approximately 90% of claims, minimizing manual administrative tasks and expediting patient access to treatment. However, challenges remain-particularly in integrating AI tools with legacy EMR infrastructures, managing language and documentation diversity, and mitigating errors arising from algorithmic misinterpretation. Addressing these limitations is essential to ensure reliability, interoperability, and clinician trust in AI-assisted prior authorization systems.

AI-powered medical billing in Saudi Arabia

AI-driven medical coding and billing in Saudi Arabia presents significant business opportunities in the healthcare sector, especially for organizations looking to streamline operations, reduce costs, and drive sustainable growth. By integrating AI solutions into the revenue cycle, healthcare enterprises can achieve faster billing processes, ensure compliance with regulatory standards, and enhance overall financial performance. Several organizations have developed AI-assisted medical coding platforms aimed at improving compliance and operational efficiency. For instance, ANOVA Health (Riyadh, Saudi Arabia; anovahealth.sa/coding) provides automated coding and mapping tools designed to align with national healthcare standards and regulatory frameworks. Future studies should focus on quantifying real-world performance and generalizability to ensure these technologies deliver consistent, evidence-based outcomes rather than solely business-oriented benefits.

Their services focus on accurate data capture and coding system implementation, which are essential for effective revenue cycle management. AI-powered tools, such as those offered by AI Code (TachyHealth, Dubai, United Arab Emirates), expedite the coding process by automatically converting medical documents into precise codes. Beamtree (Beamtree Holdings Ltd, Redfern, New South Wales, Australia) has entered Saudi Arabia's private healthcare sector through a partnership with Dr. Sulaiman Al Habib Medical Group (HMG) (Riyadh, Saudi Arabia), which provides AI-driven clinical coding technology for enhancing data quality and improving hospital coding efficiency. Athir RCM (Athir, Jeddah, Makkah Al Mukarramah; athir.com.sa) provides an AI-driven solution that digitizes the medical insurance cycle by capturing clinical and financial transactions within a centralized platform. The system facilitates the documentation cycle for service providers while offering medical coding and billing services, contributing to improved revenue cycle management. These advancements reflect Saudi Arabia’s growing adoption of AI and automation to streamline medical coding, minimize administrative burdens, and enhance overall healthcare efficiency. Success stories from companies like ANOVA Health, Beamtree, TachyHealth, and Athir RCM attract both local and international investors seeking technologically advanced healthcare ventures [41-44]. This growing investment has the potential to further stimulate research and development, fostering a continuous cycle of innovation that strengthens the healthcare ecosystem in Saudi Arabia.

AI and fraud in medical billing and coding

Medical billing fraud poses serious challenges to healthcare systems, resulting in financial losses and compromising institutional integrity [15]. AI has notably advanced fraud detection in medical billing by enabling systems to analyze extensive datasets, recognize complex patterns, and flag suspicious claims for further investigation. ML algorithms are particularly effective in identifying anomalies and irregular billing behaviors that may indicate fraudulent activity. For example, peer-reviewed studies have reported reductions of up to 20-30% in fraudulent claims following the implementation of AI-based analytics, underscoring their potential for financial protection and operational transparency in healthcare billing systems. These systems can detect unusual billing trends, inflated charges for procedures, or claims for services that were not actually provided. By cross-referencing EMRs with billing codes, AI can identify inconsistencies, preventing overbilling and fraudulent claims while ensuring compliance [45]. Additionally, integrating ML with NLP allows AI to analyze clinical visit records, compare documented services with billed procedures, and pinpoint mismatches in billing documentation. AI not only detects known fraudulent schemes but also uncovers subtle irregularities that may indicate previously unidentified fraud patterns. This proactive approach is especially valuable in addressing sophisticated fraud tactics that traditional rule-based systems may fail to detect. Furthermore, real-time claim audits, predictive analytics, and blockchain technology improve data transparency, enhance accuracy, and reduce the likelihood of billing fraud. By implementing AI-powered fraud detection, healthcare organizations can strengthen fraud prevention, minimize financial risks, and uphold the integrity of their billing operations. The adoption of AI in fraud prevention also promotes accountability and regulatory compliance, fostering trust within the healthcare industry and ensuring financial sustainability [45].

Positive impact of AI in medical billing

Enhancing billing efficiency with AI requires identifying specific areas for improvement, such as automating CPT code classification and converting operative notes into accurate billing codes. Collaborating with data scientists to develop supervised ML models or leveraging open-source AI solutions can facilitate the creation of effective algorithms [1]. AI can also be utilized to detect underbilling by comparing AI-generated CPT codes with those assigned by the billing department, ensuring accuracy and preventing potential revenue loss. Furthermore, incorporating AI into prior authorization processes can optimize clinical workflows, reduce administrative burdens, and improve overall efficiency in medical billing operations [34].

As mentioned earlier, the incorporation of AI in medical billing is transforming the efficiency and accuracy of billing operations within healthcare organizations. AI-driven systems utilize ML algorithms and automation to streamline workflows, increase productivity, and enhance financial outcomes [36]. By automating claims processing and coding, AI reduces administrative workload, enabling healthcare professionals to concentrate on patient care and more complex tasks (Figures 2, 3). Additionally, AI minimizes billing errors by identifying discrepancies and flagging questionable claims before submission, reducing financial losses and facilitating a smoother reimbursement process [15].

AI also plays a crucial role in fraud detection by analyzing extensive billing datasets, recognizing suspicious patterns, and allowing timely interventions to maintain financial integrity [46,47]. Another key advantage is revenue optimization, which refers to the strategic use of AI to maximize financial returns by analyzing coding accuracy, payer behavior, and reimbursement trends. AI systems identify missed charges, underpayments, and coding inefficiencies, helping healthcare providers recover lost revenue and reduce claim denials. By improving efficiency, minimizing errors, detecting fraud, and ensuring compliance, AI enhances revenue cycle management and promotes the financial sustainability of healthcare organizations.

Impact and challenge of AI in medical billing

The use of AI in medical billing holds great promise, but it also brings challenges and ethical concerns that must be carefully addressed. One primary issue is data privacy and security, as AI systems require access to sensitive patient information [48]. Healthcare organizations must enforce strict data protection protocols, including encryption, secure infrastructure, and compliance with regulatory standards, to prevent breaches and maintain patient trust. Additionally, institutions are increasingly developing proprietary AI systems with built-in privacy safeguards and localized data processing to minimize exposure risks and ensure greater control over sensitive patient information. Another critical consideration is bias and fairness in AI algorithms, as training data or algorithm design flaws can lead to inequitable billing practices. Ensuring diverse and representative datasets, combined with thorough validation processes, helps mitigate bias and promote fairness. Transparency and explainability are also essential to building trust in AI-driven billing. While complex algorithms can obscure decision-making, adopting interpretable ML models provides clearer insights into billing outcomes [49].

Legal compliance is another significant factor, requiring AI systems to conform to established regulations governing billing and patient rights. Strong monitoring mechanisms must be in place to detect and resolve vulnerabilities. Additionally, comprehensive training programs are necessary to equip healthcare teams with the skills needed to effectively manage AI systems [50]. Lastly, maintaining professional accountability is crucial, as human oversight ensures that AI-driven billing decisions align with ethical standards and industry regulations [49]. By proactively managing these challenges, healthcare organizations can implement AI responsibly, enhancing billing efficiency while upholding principles of compliance, equity, and transparency.

## Conclusions

The integration of AI into medical billing represents a transformative shift in healthcare administration, enhancing accuracy, efficiency, and financial performance. However, beyond its advantages, this review highlights critical challenges that must be addressed to ensure sustainable implementation. Barriers such as interoperability with legacy systems, high initial investment costs, and limited workforce training remain major obstacles to widespread adoption. Strengthening collaboration among policymakers, healthcare providers, and technology developers is essential to building scalable, secure, and interoperable AI frameworks.

In the context of Saudi Arabia, AI-based billing solutions have shown promise in improving financial transparency, reducing claim delays, and increasing coding accuracy. Linking these developments to national initiatives underlines the potential for AI to support healthcare reform and economic diversification. Future research should focus on evidence-based pilot programs, cost-effectiveness analyses, and cross-country evaluations to measure real-world impact. Predictive analytics and deep learning tools could play a pivotal role in advancing automation while maintaining compliance and patient data protection. A balanced approach that embraces innovation while addressing practical barriers will ensure that AI-driven medical billing evolves responsibly and effectively across diverse healthcare systems.
